# Malaria in China: a discourse-historical perspective

**DOI:** 10.3389/fmed.2025.1590518

**Published:** 2025-07-02

**Authors:** Peng Miao

**Affiliations:** College of Foreign Languages, University of Shanghai for Science and Technology, Shanghai, China

**Keywords:** ague, malaria, plasmodium, parasite, anopheles, history of medicine, China

## Abstract

The translation, transmission, and re-conceptualization of malaria in late Qing and Republican China exemplifies how knowledge on an ancient disease is reshaped through linguistic and cultural mediation. This article analyzes diverse textual medical sources, namely English-Chinese dictionaries (1830s-1900s) and vernacular newspapers and periodicals, to trace and observe the lexical journey of “ague” and “malaria” into the Chinese domain as “nueji” (瘧疾/疟疾) and “zhangqi” (瘴氣/瘴气). Three phases of conceptual transfer are identified: first, early missionary dictionaries (1822–1860s) prioritized symptom-based translations (e.g., faleng 發冷/发冷, chills); second, the 1870s-1920s witnessed terminological competition between nueji and zhangqi, reflecting clashes between traditional Chinese etiology and western theories; third, by the 1930s-1940s, nueji became dominant through institutional standardization, while western parasitological frameworks were selectively assimilated, as “*Plasmodium*” was lexicalized as “nueyuanchong” (瘧原蟲/疟原虫), yet the mechanism of “immunity” remained unexplained in Chinese medical discourse. This process was formed by intra-medical debates: while western-trained practitioners weaponized microscopy to validate *Plasmodium* as a pathogen, traditional healers reframed it through local cosmology. Newspaper and periodicals served as contested epistemic spaces, where terms like “weichong” (微蟲/微虫) and “jishengchong” (寄生蟲/寄生虫) mirrored public struggles to reconcile western knowledge with local beliefs. This article demonstrates that disease introduction transcends lexical substitution, acting as a battlefield for different medical discourses in China’s medical modernization.

## 1 Introduction

In recent years, translation studies have increasingly drawn upon multidisciplinary approaches, integrating perspectives from various related disciplines. This convergence is attributed to the multifactedness and natural capability of translation to incorporate diverse viewpoints from different fields (1). Such a multidisciplinary nature of translation studies has been extensively discussed (2, 3), and translation history, among various branches of translation studies, has become a vibrant interdisciplinary arena (4). Histories of translation, “a flowering” of which has been seen in recent years (5), is now considered “commensurable” with histories of science, both having “a desire to move toward ‘transnational’ or ‘global’ history” (6). In such a trend, within China’s academic landscape, this revival of translation historiography (7) has also catalyzed innovative dialogs with the history of scientific disciplines. Translation historiography traces conceptual and terminological changes in different scientific disciplines along the history, extending and enriching its research content, and provides studies on history of these disciplines with new perspectives. In the field of medicine, an increasingly strong link has been established between translation and the dissemination and construction of medical knowledge in history of cross-cultural transmission of Western medicine to China (8–10).

At the turn of the twenty-first century, the cultural turn of Western historiography extended the socio-cultural dimension of research on medical history, and seminal studies emerged in the fields of cultural and social history of medicine and health in China (11–13), among which the history of diseases became a hot topic (14). The history on the translingual practice of Western disease concepts in a West–East direction then became a significant object of research on the Chinese medical history. A typical example was provided by Andrews (11), who systematically studied the introduction of the concept of tuberculosis and the germ theory from the West to modern China and their “assimilation” in this country. “Creating a Modern Medical Vocabulary in Chinese,” as an early section of that paper, forecasted the important role of research on history of term translation in the field of disease historiography in China.

As the multidisciplinary feature of translation history came to notice, and the perspective of translation history in the field of medical history was acknowledged, new possibilities of writing the histories of the emergence, promotion, and reception of disease concepts from Western medicine in modern China emerged. The translation histories of disease concepts transcend that of terminological shifts and conceptual transference, and include histories of introduction, re-production, and reception of systematic medical knowledge. As such, research on the translation, dissemination, and reception of concepts and knowledge of certain diseases is no longer restricted to the translation practices of relevant terms; instead, such a process should be viewed as conceptual movement, which begins with the translation practices involving a source language and a target language at the textual level, and then proceeds to knowledge construction from discourses to ideas at the cognitive level.

The translingual practices of disease concepts begin with the translation of disease names. The nomenclature of diseases makes possible the domestication of the concepts behind the names, a factor promoting the dissemination and reception of these concepts. The translation of disease names is not only about the coinage or selection of the terms for the diseases; it also involves explanations for the disease concepts to be translated and introduced. Therefore, such a process is not merely one that involves the translation of several terms. It is a “travel” of systematic knowledge among different cultures. To demonstrate its complexity, the current article adopts the translingual journey of malaria (nueji 瘧疾/疟疾 in Chinese) as a case study. Malaria is more than a case of translingual practice; instead, it epitomizes the epistemic tensions underlying China’s medical modernization.

A reconstruction of malaria’s lexical journey in Chinese medical discourse helps demonstrate how cross-cultural language and information exchanges mediated the assimilation of Western biomedicine while preserving traditional etiologies. It is also an attempt to make innovative contributions to paleopathological studies in three aspects. First, a cross-disciplinary framework integrating discourse studies with medical history is adopted, to reveal how diverse textual medical sources shaped disease perception. Second, a cross-temporal discussion is led, covering both the historical development of malaria in China and the present difficulties in malaria prevention and treatment; it resonates with the main basis of paleopathological research—“the understanding of disease in its modern context” (15). Third, a cross-spatial dialog might be triggered, as the findings of this research bridge existing works on malaria in the western context, such as Boualam et al.’s historical and paleopathological investigations of malaria in Europe (16), offering a non-western perspective, therefore enriching global narratives of disease historiography.

In this article, the materials were meticulously selected and the method was carefully designed to ensure its scientific rigor and comprehensiveness. The materials primarily consist of two categories, English-Chinese dictionaries compiled during the late Qing and Republican periods, and newspaper and periodical articles from the same era. As for the dictionaries, an exhaustive search of keywords (“ague” and “malaria”) was conducted by using “The English-Chinese Dictionary Database” in “Modern History Databases” (Institute of Modern History, Academia Sinica); as for the articles in newspapers and periodicals, terms like “nue,” “nueji,” and “zhangnue” were used to retrieve malaria-related texts from CNBKSY (abbreviation of Quan Guo Bao Kan Suo Yin 全國報刊索引/全国报刊索引), a database of Shanghai Library containing newspapers and periodicals published in China since 1833. It was ensured that the texts selected were directly relevant with the objectives of this article, and capable of providing a comprehensive understanding of malaria in the Chinese medical discourse. Methodologically, this article studies the introduction of malaria to China within a thematic historical framework. Specifically, the dictionary entries were meticulously analyzed for a detailed account of how “malaria” was translated into the Chinese language, whereas the newspaper and periodical articles were carefully studied for a further explanation on how “malaria” was explained to the recipients of the Western knowledge.

## 2 Malaria in Western and Chinese medical history

Throughout human history, malaria has emerged as a significant disease concept. It appeared in ancient writings of both Western and Chinese civilizations. In the Western medical tradition, malaria’s origins could be traced back to classical Greek and Roman medical literature. Hippocrates is believed the first physician to document a malarial paroxysm (17). During his time, the disease was broadly referred to as “fever.” Subsequently, in ancient Rome, it was described as “intense burning heat” (18). The vagueness of its terminology in the ancient times made it extremely challenging and uncertain to distinguish malarial fevers from other types of fevers (19). The term “malaria” was derived from the Italian words “mal’aria,” literally meaning “bad air” (or “evil air,” or “corrupted air”), and was first used in Francesco Puccinotti’s book *Storia delle febbri intermittenti di Roma* published in 1838 (17). This term revealed an association of the disease with environmental factors, such as swamps and marshes, in traditional Western medicine. Today, malaria is no longer recognized as a disease caused by “bad air”; instead, it is understood as an infectious disease caused by “obligate intracellular protozoa of the genus *Plasmodium*” and usually transmitted through bites of “infected anopheline mosquitoes” (20).

In Chinese classics, mentioning of this disease could also be observed. For instance, in *Huang Di Nei Jing* (The Yellow Emperor’s Inner Classic 黃帝内經/黄帝内经), the following dialog between Huang Di and Qi Bo, excerpted from Chapter 35 “Discourse on Malaria,” was about the relationship between wind and malaria:

Huang Di: “Now, wind and malaria resemble each other, and they belong to the same category. However, only the wind is present permanently, while the malaria may rest at [specific] time. Why?”

[Original: 夫风之与疟也, 相似同类, 而风独常在, 疟得有时而休者, 何也?]

Qi Bo: “The wind qi stays at its location, hence it is permanently present, while the malaria qi following the conduits and network [vessels moves] deep to strike from the inside. Hence, when the guard qi responds, then it is active.”

[Original: 风气留其处, 故常在, 疟气随经络, 沉以内薄, 故卫气应乃作.]

This is a translation by P. Unschuld (21). Malaria, in the original text, was referred to as “nue” (瘧/疟).

In *Shuo Wen Jie Zi* (説文解字/说文解字), “nue” was explained as intermittent chills and fever; in *Shi Ming* (釋名/释名), “nue” meant cruel treatment of a person, implying that it tortured its patient with both chills and fever. These examples from Chinese classics help illustrate that the concept of malaria in China was not completely loan from the Western medicine. As such, it becomes necessary to clarify that the translation of malaria discussed by the current article refers to that in Late Qing and Republican China, during the dissemination of Western medical knowledge in the Chinese context.

In the Western medical tradition, malaria had multiple synonyms - ague, paludisme, Wechselfieber, Dardag Kolle, triasuchka, accessia, marsh fever, Lord John’s fever, among many others (17). Similarly, in Traditional Chinese Medicine (TCM), malaria was referred to by diverse terms, including shiyi (濕疫/湿疫), wenshiji (溫濕疾/温湿疾), zhangqi (瘴氣/瘴气), zhangli (瘴癘/瘴疬), guinue (鬼瘧/鬼疟), and zhangnue (瘴瘧/瘴疟), as was summarized by Fan Xingzhun (範行准/范行准) (22).

**Figure 1:**
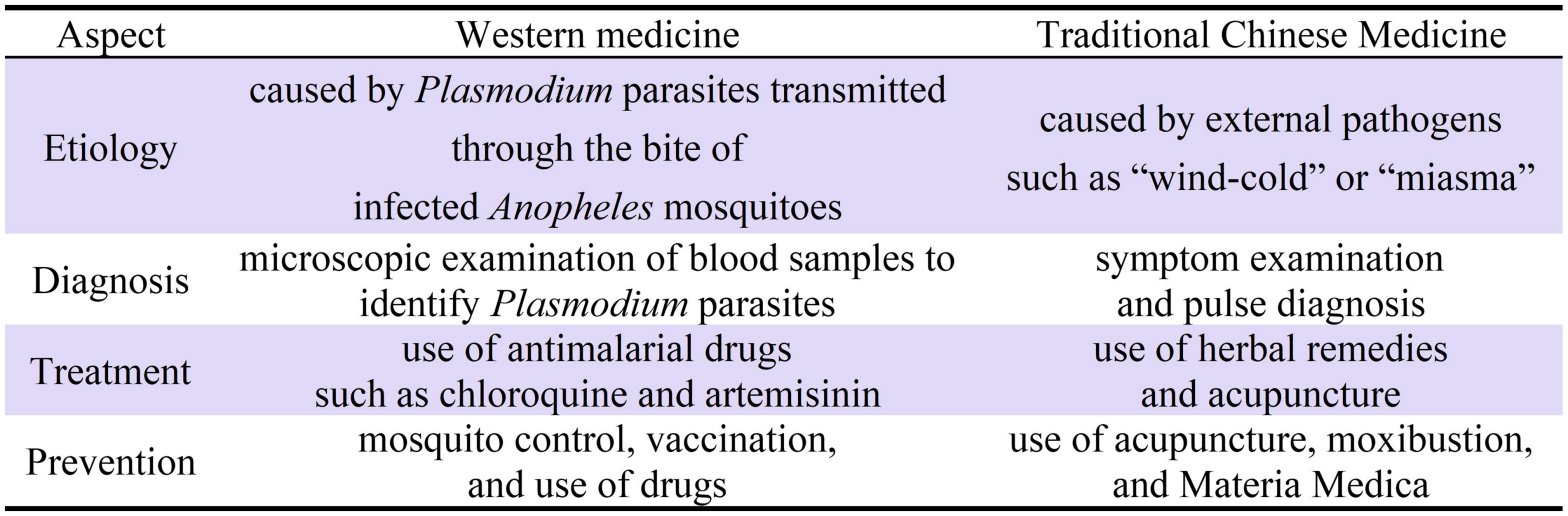
A comparison between the concepts of Western medicine and traditional Chinese medicine in the context of malaria.

The Chinese term “nueji” currently used implies knowledge translated from the West in the 19^th^ and 20^th^ century. The translation of malaria in China provided a platform of conversation and interaction for Western medicine and TCM. Subsequently, it led to the merge of the Western medical concept of malaria and the TCM concept of “nue.” In other words, “nueji” in the Chinese medical discourse today is a combination of the two. A comparison between the concepts of Western medicine and TCM in the context of malaria is presented in Figure 1.

In his renowned work illustrating disease perceptions in Chinese history, *A New History of Diseases in China*, Fan explained that “nueji” was one of the oldest acute contagions in the country’s history. Early in Zhou Dynasty (1,046 A. D.–771 A. D.), the Chinese linked the disease with the climate; during 770 A. D. and 221 A. D., the Chinese were aware of malaria symptom of intermittent chills and fever, and its patients’ sufferings during the sweating stage. After 221 A. D., the belief that the disease was caused by “wind-cold” (fenghan 風寒/风寒) began to prevail, and the geographical factor became also covered in its etiology (22). Evidently, TCM had a powerful explanatory system concerning malaria’s etiology. Also, its treatment was early discussed in *Zhou Hou Bei Ji Fang* (Emergency Prescriptions kept in one’s Sleeve 肘後備急方/肘后备急方) by Ge Hong (葛洪), who was the first in medical history to recommend the use of qing hao (青蒿) for the treatment of “nue” (23), later shedding light upon Tu Youyou and her colleagues’ discovery of artemisinin (24). As such, TCM had an ineligible influence on the introduction of the Western medical concept of malaria to China nearly 2000 years later. Such an influence was first revealed by the equivalents to “malaria” and “ague” provided by the early missionary dictionaries in China.

## 3 Translating malaria

### 3.1 Dictionary translations of “ague” and “malaria” since late Qing

**Figure 2:**
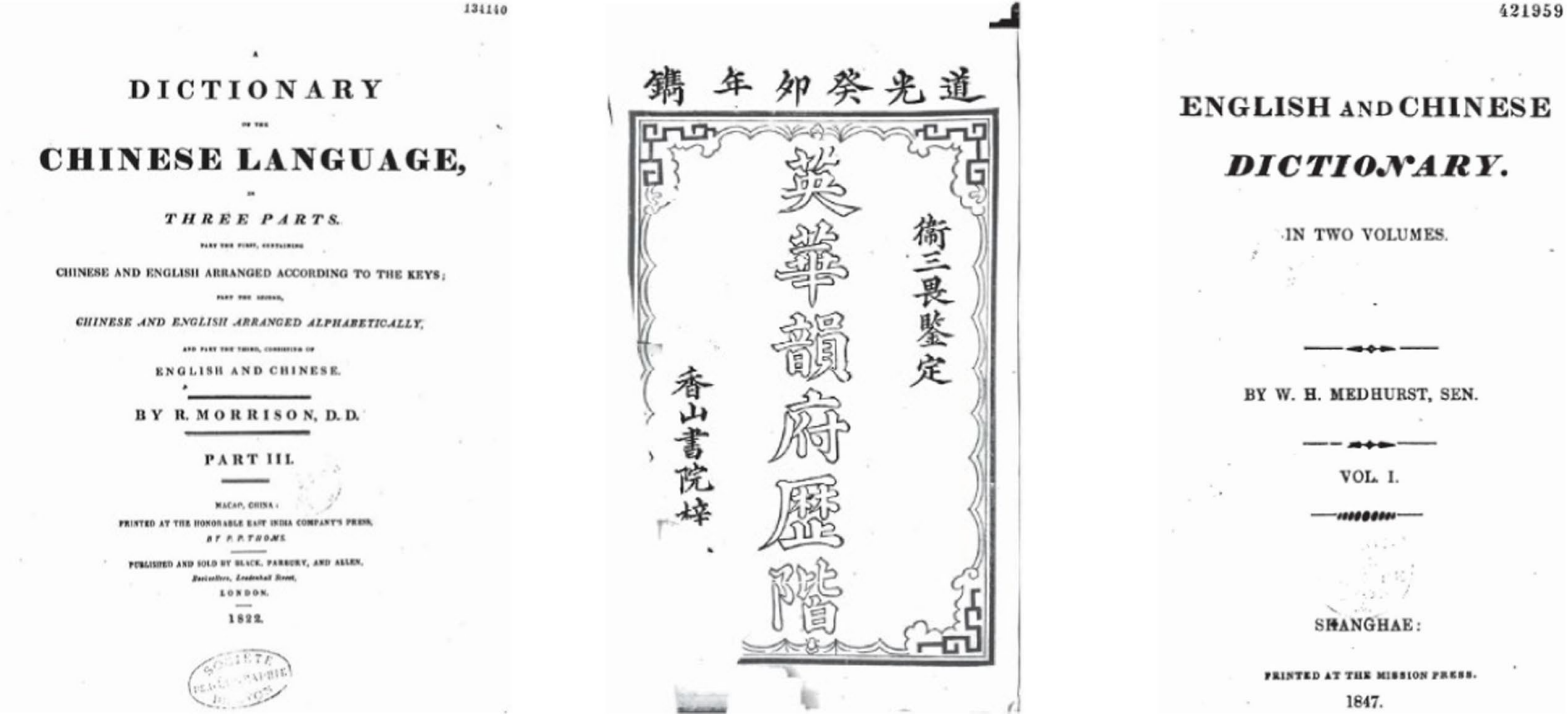
The most well-known English-Chinese Dictionaries in the first half of the nineteenth century. Source: “The English-Chinese Dictionary Database” in “Modern History DataBases” (Institute of Modern History, Academia Sinica). Reproduced with permission from Institute of Modern History, Academia Sinica.

Both “malaria” and “ague” are common terms denoting this ancient disease in English. The former remained the predominant designation for malaria in medieval English medical discourse, persisting until the identification of *Plasmodium* as the pathogenic genus in the late nineteenth century (16). The Chinese people encountered these two terms in the mid-nineteenth century, when a series of English-Chinese dictionaries compiled by foreign missionaries working in this country were published. Before 1850, *A Dictionary of the Chinese Language in Three Parts by Morrison* (1822), *An English and Chinese Vocabulary, in the Court Dialect by Williams* (1844), and *English and Chinese Dictionary* by Medhurst (1847–48) were the most well-known English-Chinese dictionaries (Figure 2). In all of these three dictionaries, an entry of “ague” was included, while “malaria” was not.

**Figure 3:**
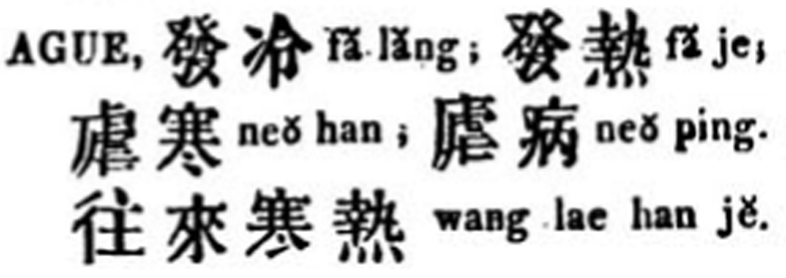
“Ague” in Morrison’s dictionary. Source: “The English-Chinese Dictionary Database” in “Modern History DataBases” (Institute of Modern History, Academia Sinica). Reproduced with permission from Institute of Modern History, Academia Sinica.

The earliest record of the entry “ague” was provided by Morrison’s dictionary, where the term was translated into “faleng 發冷/发冷,” “fare 發熱/发热,” “nuehan 虐寒,” “nuebing 瘧病/虐病,” and “wanglai hanre 往來寒熱/往来寒热” (Figure 3) (25). Among these translations, faleng, meaning chill, fare, meaning fever, and wanglai hanre, meaning intermittent chills and fever, all denote the symptoms of the disease. Faleng and fare are even antonymous, while wanglai hanre is in accordance with the traditional local account of the disease’s symptom. Nuehan and nuebing both contain the character nue, which is a component of the disease’s contemporary name in the Chinese language. William’s dictionary translated “ague” into one single term “faleng,” avoiding the possible ambiguity caused by the concurrence of faleng and fare as its equivalents.

**Figure 4:**
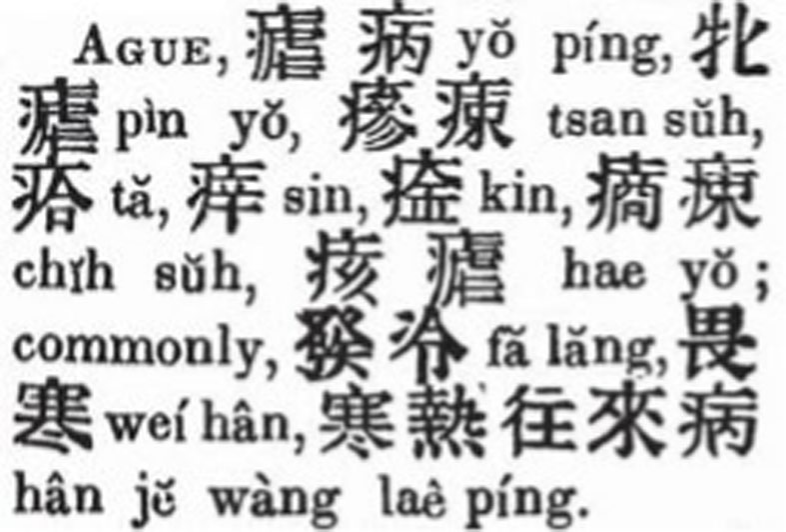
Eleven equivalents of “ague” in Medhurst’s dictionary. Source: “The English-Chinese Dictionary Database” in “Modern History DataBases” (Institute of Modern History, Academia Sinica). Reproduced with permission from Institute of Modern History, Academia Sinica.

**Figure 5:**
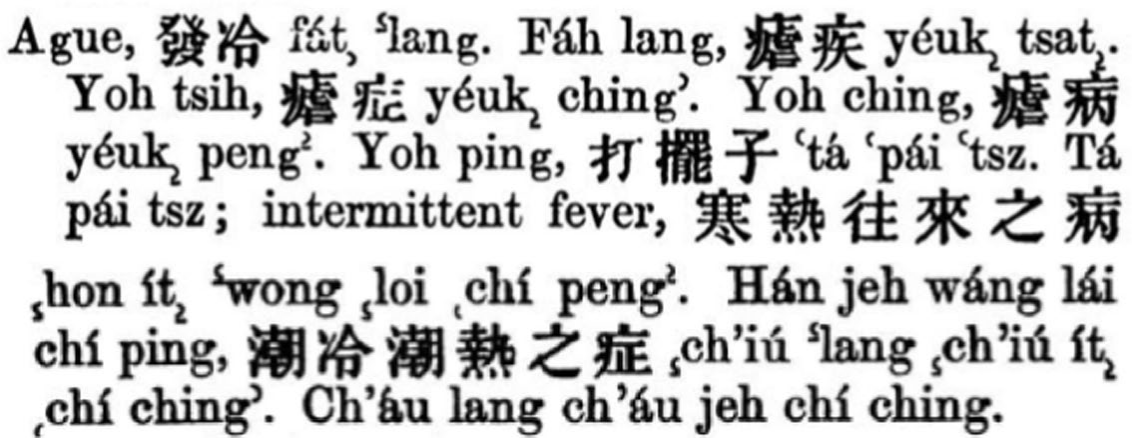
Seven equivalents of “ague” in Lobscheid’s dictionary. Source: “The English-Chinese Dictionary Database” in “Modern History DataBases” (Institute of Modern History, Academia Sinica). Reproduced with permission from Institute of Modern History, Academia Sinica.

Medhurst’s dictionary provided the richest translations of the term among the three (Figure 4) (26). *English and Chinese Dictionary with the Punti and Mandarin Pronunciation* compiled by Lobscheid and published during 1866 and 1869 included “dabaizi 打擺子/打摆子,” a folklore term for malaria meaning “bodily shivering,” and “nueji,” among the translations of “ague” for the first time (Figure 5); the entry of “malaria,” translated into “zhangqi 瘴氣/瘴气” and “lanzhang 嵐瘴/岚瘴,” was also included in this dictionary (27).

Since then, “ague” and “malaria,” as two separate entries having completely different translations, coexisted in English-Chinese dictionaries for over 40 years. The equivalents of “ague” mainly denoted the symptoms of the disease, or contained the character “nue 瘧/疟,” whereas the equivalents of “malaria” mainly contained the character “zhang 瘴.” For instance, in *Vocabulary and Hand-book of the Chinese Language* compiled by Doolittle (1872), “ague” was translated into “faleng,” “nuebing,” “fanuezi 發瘧子/发疟子,” and “hanre wanglai bing 寒熱往來病/寒热往来病,” while “malaria” was translated into “shanlan zhangqi 山嵐瘴氣/山岚瘴气” and “fudu zhiqi 腐毒之氣/腐毒之气” (28). The first and fourth equivalents of “ague” denoted the symptoms, and second and third contained “nue”; the first equivalent of “malaria” contained “zhang,” and both explained the concept as pathogenic qi that causes diseases.

**Figure 6:**
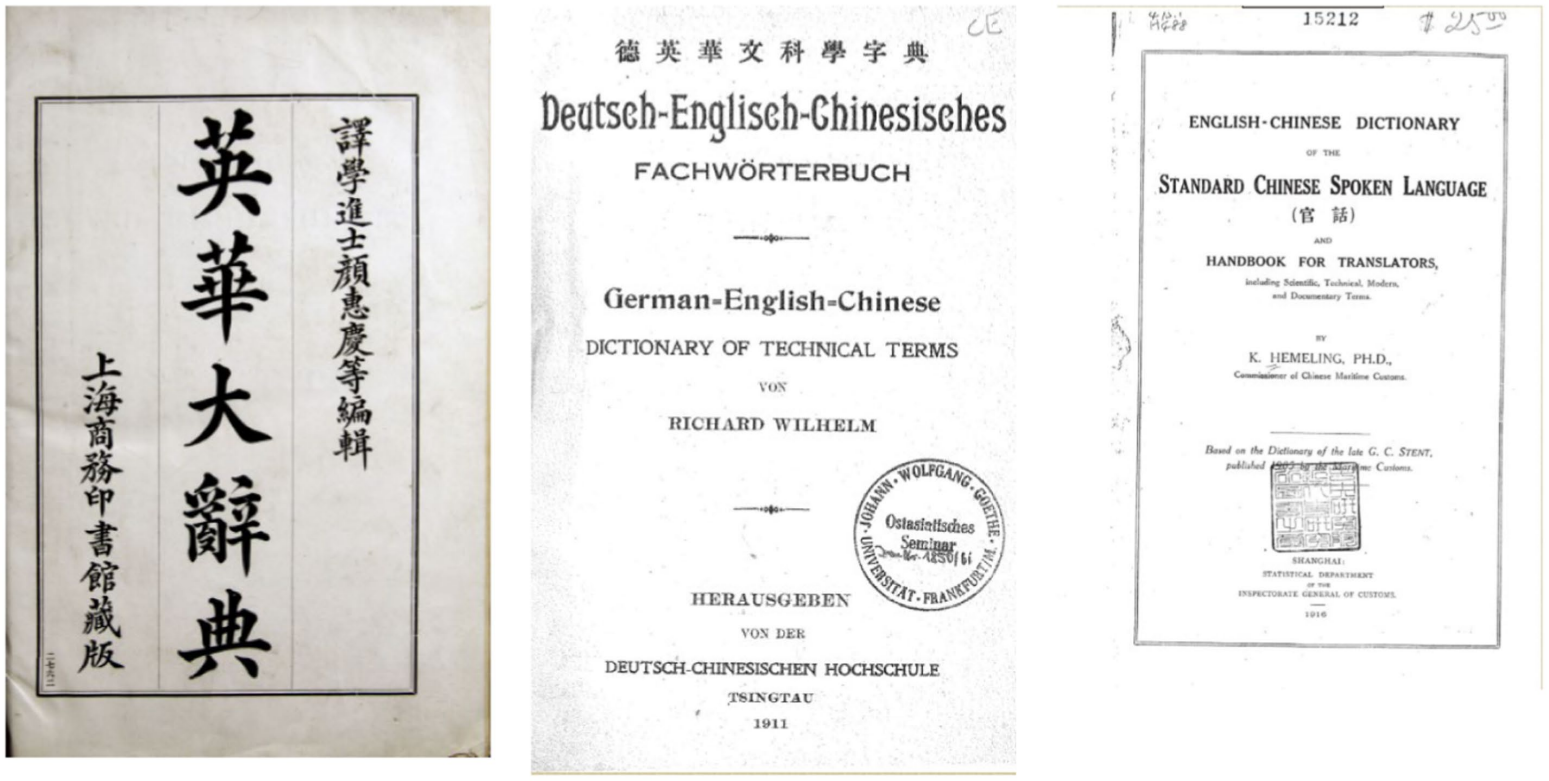
Yen’s (left), Wilhelm’s (middle), and Hemeling’s (right) dictionaries. Source: “The English-Chinese Dictionary Database” in “Modern History DataBases” (Institute of Modern History, Academia Sinica). Reproduced with permission from Institute of Modern History, Academia Sinica.

**Figure 7:**
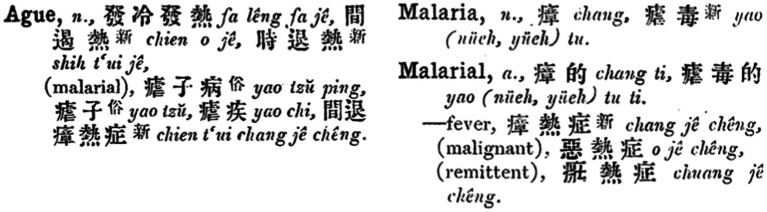
“Ague” and “malaria” in Hemeling’s 官话. Source: “The English-Chinese Dictionary Database” in “Modern History DataBases” (Institute of Modern History, Academia Sinica). Reproduced with permission from Institute of Modern History, Academia Sinica.

Entering the early twentieth century, several influential English-Chinese dictionaries were consecutively published (Figure 6). In 1904, *Technical Terms* (術語辭匯/术语辞汇) compiled by C. W. Mateer was published in Shanghai. This lexicon was the earliest to link “ague” and “malaria” by including “nuedu 瘧毒/疟毒 (literally meaning the toxin or poison of nue)” as one of the translations of “malaria” (29), and thus the character nue became shared by both “ague” and “malaria.” Yen Wei-Ching’s dictionary, *An English and Chinese Standard Dictionary* (1908), also showed such a link between the two (30). *English-Chinese Dictionary of the Standard Chinese Spoken Language* (官話/官话) *and Handbook for Translators* (1916) by Hemeling further included “malarial ague” within the entry of “ague,” and translated “malaria” into “zhang, nuedu” (Figure 7) (31). Wilhelm’s *Deutsch-Englisch-Chinesisches Fachwörterbuch* (1911) was slightly different: “malaria” was translated into “zhangqi, nueji,” whereas “ague” was absent (32).

The equivalence between “nueji” and “malaria” was also gradually formed during the 1920s, when the General Committee for Scientific Terminology, a unit subordinate to China Medical Missionary Association, actively censored and unified the Chinese terms for the introduced scientific concepts. The committee published eight booklets for the unification of medical terms, among which the sixth volume published in July 1921 included the entries of “ague” and “malaria,” and translated both into a single character: “nue.”

**Figure 8:**
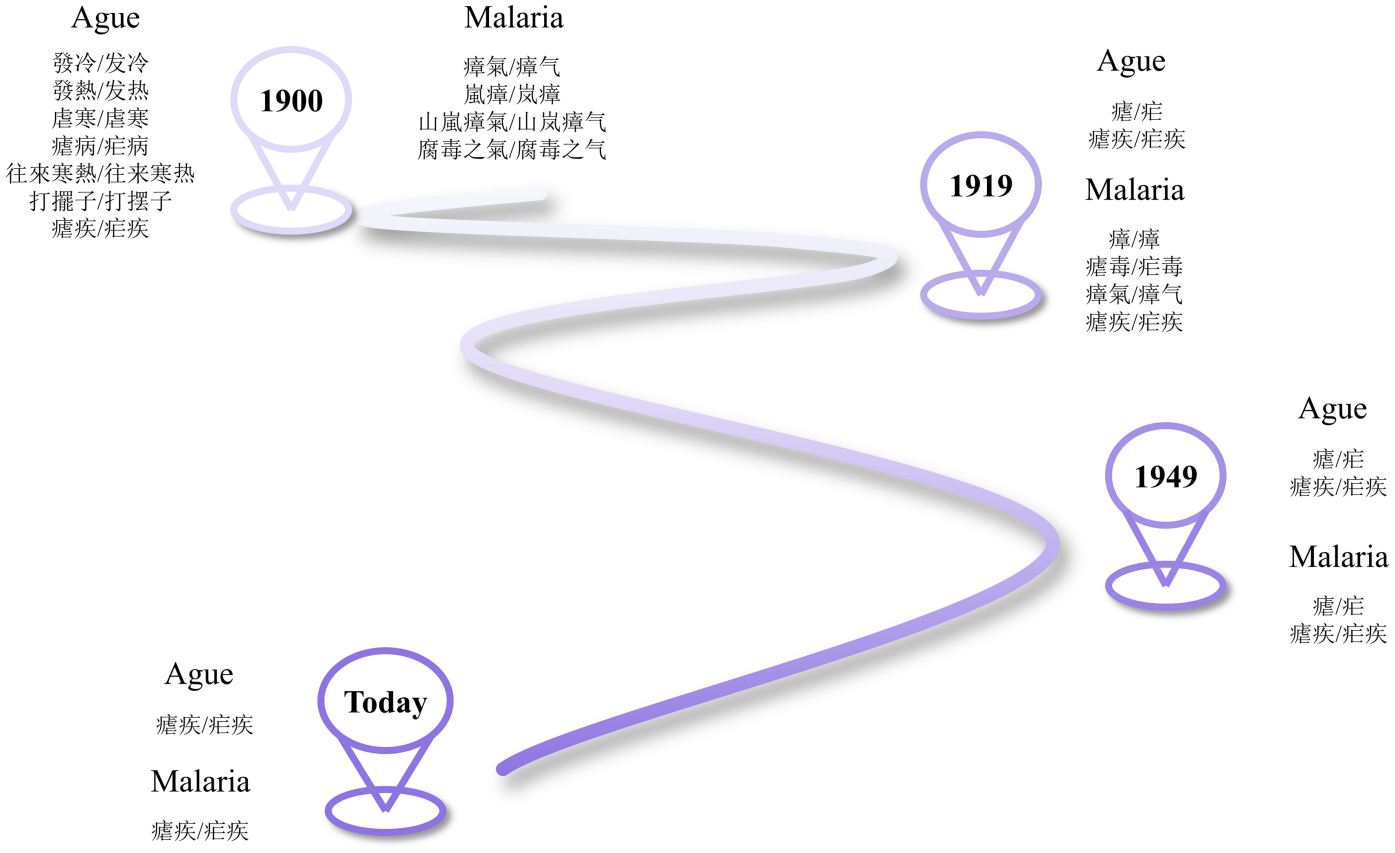
Evolution of translations of “ague” and “malaria”.

In the 1930s and 1940s, the translations of “ague” and “malaria” became gradually unified and close to the committee’s versions. For example, *English-Chinese Modern Medical Dictionary* (1949) translated both into “nue,” with the entry “malaria” followed by a series of relevant terms, including “malariologist,” translated into “nueji zhuanjia 瘧疾專家/疟疾专家,” and “malariology,” translated into “nueji xue 瘧疾學/疟疾学,” demonstrating the steadiness of equivalence between “malaria” and “nueji” (33). Figure 8 shows how translations of “malaria” and “ague” evolved in the 19^th^ and the first half of the 20^th^ century.

### 3.2 Newspaper accounts on “nueji” and “zhangqi” since the turn of the twentieth century

By the end of the nineteenth century, the Chinese term “nueji” had begun to appear in articles published in different types of newspapers. The timepoint when “nueji” and “zhangqi” converged shown by newspaper resources is identical to that shown by dictionaries. In this section, the use of “nueji” and “zhangqi” in modern newspapers and the convergence of the two are to be observed and analyzed, and in the next two sections, the shift of ideas and change of cognition behind the changing terms are to be discussed.

The use of “nueji” in newspapers originated in the late 1860s. In an article entitled “nueji jiechuang zhiyuan (etiology of malaria and scabies 瘧疾疥瘡之原/疟疾疥疮之原),” it was introduced that the foreigners investigated the etiology of malaria and proved that “nuezheng (a synonym of nueji)” was indeed caused by “shidi” (literally meaning “marshes” 濕地/湿地) (34).

Entering the twentieth century, the number of newspaper articles on “nueji” gradually rose, and as is manifested by data from CNBKSY, it peaked in the 1930s (over 700). Till the founding of People’s Republic of China, the discussions on “nueji” in medical and popular newspapers and periodicals mainly focused on the media of its transmission, its etiology, its treatment, its prevention, and the differences between theories of Western medicine and TCM about this disease. Meanwhile, less discussion on “zhangqi” could be observed.

**Figure 9:**
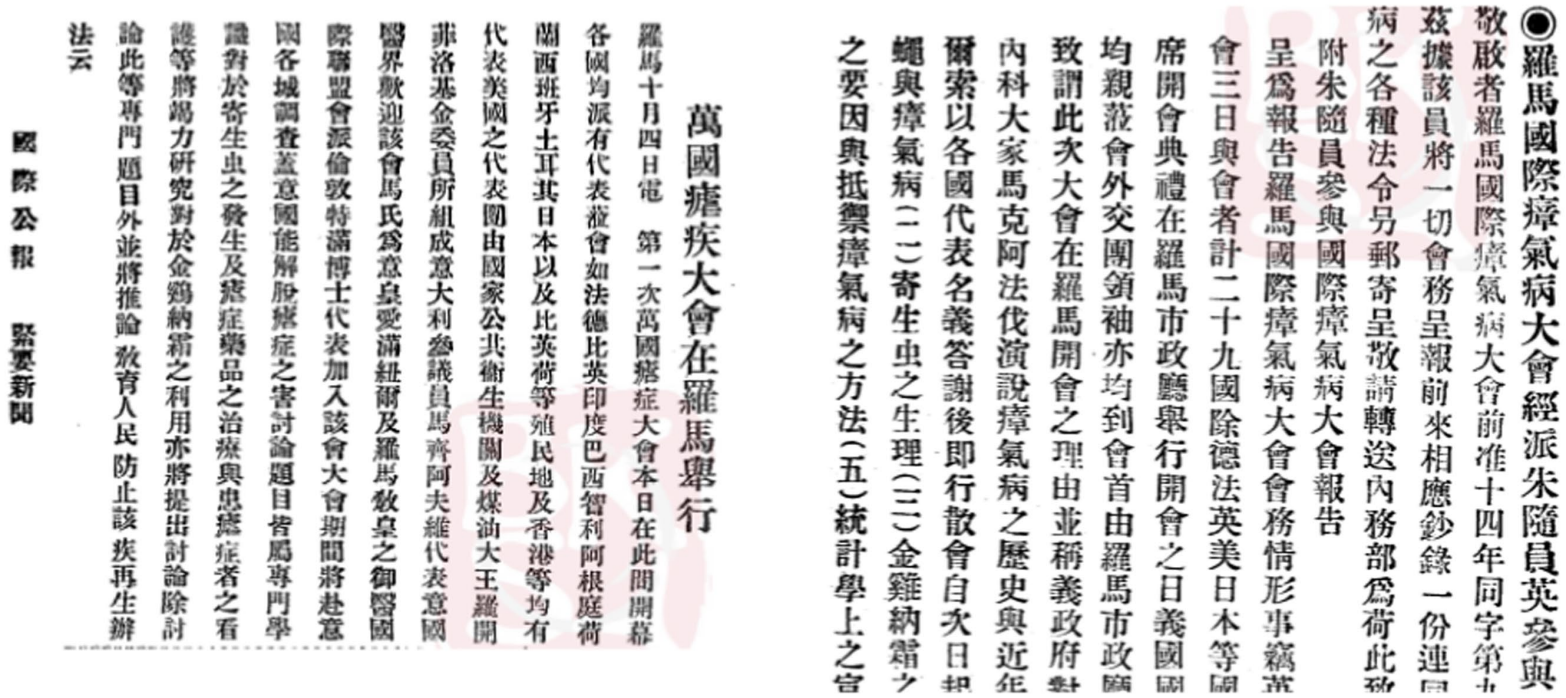
Articles denoted International Congress for Malaria with phrases containing “*nueji*” and “*zhangqi*”. Source: “CNBKSY. Reproduced with permission from CNBSKY, Shanghai Library.

Dictionary proofs manifested that the equivalents of “ague” and “malaria” became unified in the 1940s, and such unification could be observed through the convergence of “nueji” and “zhangqi” as translated terms. The modern newspaper and periodical proofs further showed that the relation between “nueji” and “zhangqi” was clarified around 1935. Around 1924, a malarial epidemic attacked Union of Soviet Socialist Republics. Facing its spread, the First International Congress for Malaria was held in Rome, the news on which spread quickly in China through newspaper articles. The congress was denoted by different names, “wanguo nueji dahui” (the world *nueji* congress 萬國瘧疾大會/万国疟疾大会) and “luomaguo zhangqibing dahui” (the international *zhangqibing* congress in Rome 羅馬國際瘴氣病大會/罗马国瘴气病大会), in articles published in *The International Journal and Institute Record* (國際剬報/国际剬报) and *Waijiao Gongbao* (diplomatic journal 外交剬報/外交剬报), demonstrating the fact that the Chinese translation of “malaria” had not been unified (Figure 9).

In September 1935, an article entitled “zhangqibing xi exing nueji (zhangqibing is malignant nueji 瘴氣病係惡性瘧疾/瘴气病系恶性疟疾)” was published in *The Central Daily News* (中央日報/中央日报), which pointed out that “‘zhangqibing’ and ‘nuejibing’ are the same, as has been confirmed by scientific examination” (35). In the same year, the identicality between “zhang” and “nue” was also clarified by articles published in medical periodicals, such as “guisheng queren zhangqi wei exing nueji (Guangxi government confirmed that zhangqi is malignant nueji 桂省確認瘴氣為惡性瘧疾/桂省确认瘴气为恶性疟疾)” published in *Peipinger Medizinische Monatsschrift* (Peiping Medicine Monthly 北平醫刊/北平医刊) (36).

Right before the clarification of the “zhang-nue” relation, in the late 1920s, the degree of reception of the term “nueji” in Chinese was visibly higher than that two decades earlier, which could be observed by the formulation of the term’s metaphorical usage. For example, a 1927 article entitled “nueji” in *Social Welfare Tientsin Reports* (益世報/益世报) criticized the national character of the Chinese people, comparing it to a “nueji” patient suffering intermittent chills and fever (37). Another article entitled “nueji shide waijiao (nueji-like diplomacy 瘧疾式的外交/疟疾式的外交)” in *Xin Wen Bao* (新聞報/新闻报) criticized that the China-Russia relation was like a patient suffering from “nueji,” who felt very cold, hot, ill, and normal randomly without any transitional period (38). These proofs might verify to the fact that around 1930, the term “nueji” became well-established in Chinese people’s daily discourse, and its major symptoms were part of public knowledge.

## 4 Explaining malaria

### 4.1 Chinese people’s changing understanding of the etiology of malaria

The traditional Chinese and Western medicines shared the same view on the etiology of malaria. Early in the ancient Greek times, malaria was considered a type of fever of the marshes (22), while the explanation for its cause given by modern medicine dated back to approximately 150 years ago. In the late 19^th^ century, Western medicine made significant breakthroughs in understanding the cause of malaria. The pioneering studies of Louis Pasteur in France and Robert Koch in Germany accelerated the establishment of modern microbiology. On this basis, French military surgeon Alphonse Laveran made a groundbreaking discovery in November 1880: he identified the parasites in the red blood cells of malaria patients, establishing the causal relationship between the *Plasmodium* and malaria. In August 1897, British bacteriologist Ronald Ross discovered the parasites of a malaria of bird in India, and a year later, Italian zoologist Giovanni Grassi and his colleagues discovered a parasite of human malaria in an *Anopheles* mosquito. Since then, the etiology of malaria regarding *Anopheles* mosquitos as its vectors and plasmodia as its pathogens was gradually established (39, 40).

#### 4.1.1 The etiological theory on malaria from the west and the concepts of *anopheles* mosquito, parasite, and *plasmodium*

At the end of the nineteenth century, the theory of malaria being transmitted to humans by *Anopheles* mosquitoes was introduced to China approximately 10 years after it was proposed. In 1908, an article entitled “nueji yu wenzi zhi xiangguan (relation between malaria and mosquitos 瘧疾與蚊子之相關/疟疾与蚊子之相关)” was published in *The Chinese Christian Intelligencer* (通問報/通问报), explaining that Britain was free of malaria, owing to the absence of mosquitos, while Italian people suffered much from the disease, owing to the large number of the insect. This type of mosquito was called “an’afunisi wenzi (an’afunisi being a transliteration of *Anopheles*),” and it carried “nueji de chong (chong of nueji 瘧疾的蟲/疟疾的虫, literally meaning ‘worms of malaria’).” This article denoted such “chong” by the term “weichong (微蟲/微虫 literally meaning ‘micro worm’),” and introduced that under a microscope, “si weichong (dead weichong)” could be observed in “baizhu (白䏭 an archaic name of the white blood cell),” and “huo weichong (living weichong)” could be observed in “hongzhu (紅䏭/红䏭 an archaic name of the red blood cell)”; “weichong” could grow up and fission, destroying the red blood cells and causing the symptoms of malaria (41).

In the same year, “nueji zhi youlai (origin of malaria 瘧疾之由來/疟疾之由来)” by Wang Yanfu (王嚴甫/王严甫) was published by *Nong Gong Shang Bao* (農工商報/农工商报) and *Jing Ye Xun Bao* (競業旬報/竞业旬报). The article explained that malaria was transmitted to humans by a type of mosquito (called “banwen 斑蚊”) which lived in sewage and on rotten matters (“fubai zhi wu 腐敗之物/腐败之物”), from which “weijun 微菌” (an archaic name of bacteria) came into its belly; when stinging humans, the bacteria in its belly penetrated their skin through its needle-shaped mouth and destroyed the white blood cells, leading to sudden changes in blood circulation and symptoms of malaria (42).

It is noteworthy that the author’s explanation on the vector of malaria, using the term “banwen” in Chinese, is incorrect. Today, the vector of malaria, *Anopheles* mosquitoes, is referred to as “anwen” or “nuewen,” while “banwen” commonly denotes *Aedes* mosquitoes that transmit other pathogens, such dengue virus and Zika virus (43).

As is shown by these two articles, in the early twentieth century, the newspaper accounts on malaria centered on three themes: its transmission vector, pathogen, and pathogenesis.

A decade after 1908, with the popularization of the theories on mosquitos as the transmission vectors of malaria and parasites as the pathogens of the disease in China, the media of transmission of relevant knowledge extended from newspapers and periodicals within the medical field to those for popular reading, such as “yufang nueji xinshiyan (a new test on malaria prevention 預防瘧疾新試驗/预防疟疾新试验)” published in *Sin Min Pao* (新民報/新民报) in 1919, “yufang nueji fa (methods of malaria prevention 預防瘧疾法/预防疟疾法)” published by *Shen Pao* (申報/申报) in 1920, and “duwen chuanran nueji zhi yuanyin (reasons for poisonous mosquitos transmitting malaria 毒蚊傳染瘧疾之原因/毒蚊传染疟疾之原因)” published by *Xin Wen Bao* in 1922; the prevention and treatment of malaria, which was based on the theory on its etiology, was also gradually introduced to the Chinese public.

When the Western medical theory on the cause for malaria was about to be the mainstream, the second wave of large-scale debate between TCM and Western medicine (triggered by the abolishment of TCM cases) broke out, the etiology of malaria being a focus. As such, the newspaper and periodical articles on malaria around 1930 showed much suspicion of and even criticism against the theory coming from the West, as well as much effort to combine the theories of the two civilizations on the disease’s etiology to explain for its cause, which should not be ignored in discussions on the diachronic changes in Chinese people’s understanding of the etiology of malaria.

#### 4.1.2 The etiological theories on malaria in the debate between TCM and Western medicine

In the 1920s, in different types of newspapers and periodicals, articles using TCM theories to explain the cause for malaria were abundant, such as “malaliya guowei nueji bingyuan ye (is malaliya really the pathogen of nueji? 麻拉利亞果為瘧疾病原耶/麻拉利亚果为疟疾病原耶; ‘malaliya’ being a transliteration of ‘malaria’) by Shen Zhonggui (沈仲圭) in *Yixue Zazhi* (醫學雜志/医学杂志). In his article, Shen Zhonggui, an influential TCM practitioner, discussed the Western medical and TCM theories on the etiology of malaria, stating that “malaliya yuanchong (malaria parasite 麻拉利亞原蟲/麻拉利亚原虫)” entering the human blood cells was a confirmed pathogen of malaria, which was observed by Western scholars under the microscope. But he was still not convinced by this explanation; instead, he proposed that the cause for malaria should be wind-cold attacking Shaoyang (少阳 a “channel” term in TCM) (44). As the debate between TCM and Western medicine summitted, their gap in cognition on the etiology of malaria was enlarged.

In the 1930s, the TCM practitioners began to utilize the topic of malaria’s cause to question, criticize, and even oppose Western medicine. They made some attempts to promote TCM theories and suppress Western theories by drawing the readers’ attention to the shortcomings of Western etiological theories and treatment. For instance, Ji Hua proposed that though there was much research on pathology by Western medicine, its etiological explanation for malaria that the disease was caused by fission of “baozi chong” (孢子蟲/孢子虫; “baozi” meaning “spore,” and “chong” meaning “worm”) was neither confirmed nor convincing (45). Zhu Zhensheng stated that the cases where quinine failed to cure malaria owed to the incorrect explanation by Western medicine that malaria was caused by “nuejun” (瘧菌/疟菌; “jun” meaning “bacteria,” and *Plasmodium* here being mistakenly lexicalized as a type of bacterium) (46).

Conversely, the criticism against TCM by Western medical practitioners was also straightforward. A 1932 article entitled “nueji qianshuo (a brief introduction to malaria 瘧疾淺説/疟疾浅说)” published by *Yixue Zhoukan Ji* (醫學周刊集/医学周刊集) was translated from a public speech on public health in America. At the beginning of this text, the translator added, as a response to the TCM practitioners’ explanation for malaria’s cause, that malaria was not caused, as was explained by TCM, by fenghan shushi (wind-cold and summerheat dampness 風寒暑溼/风寒暑湿), or yaomo guiguai (monsters and demons 妖魔鬼怪/妖魔鬼怪), or partaking of dirty food, or being showered in turbid water and inhaling contaminated air, but caused by “nuechong” transmitted to the human body (47). Yao Jie criticized “the stubborn TCM practitioners” at that time even more directly by stating that it had been acknowledged by scholars around the world that malaria was transmitted by mosquitos, and the TCM practitioners, unwilling as they were, had to accept it (48).

The existence of *Plasmodium* was a fact proven by observation using the microscope, and thus the TCM practitioners found it impossible to fundamentally deny the claim that *Plasmodium* caused malaria. With the rise of the idea of confluence of the Chinese and Western medicines in the early years of Republican China, an expedient to merge the etiological theories on the cause for malaria of the two emerged. For instance, Huang Zushang argued that TCM and Western medical practitioners held the same view on the cause for malaria, though they used different terms (49). Wu Gong’s “nueji zhi zhongxi tan (Chinese and Western views on malaria 瘧疾之中西談/疟疾之中西谈)” was also a typical example where the Chinese and Western theories on the cause for malaria were combined, claiming that the TCM version owing malaria to “waixie (external toxin 外邪)” and the Western version owing the disease to “nuejun” were identical theories explained by different words (50). Similarly, “nueji qianshu (a brief account of malaria 瘧疾淺述/疟疾浅述),” a text merging the Chinese and Western etiological theories, owed malaria to “hanshu (cold and summerheat 寒暑)” and “baochong (胞蟲/胞虫; an archaic name of *Plasmodium*),” the former stimulating autonomous changes of the human body, and the latter invading the human body to harm its physiological mechanism (51).

As TCM declined, and the concept of “parasite” was gradually received by the Chinese people, the Western medical theory of *Plasmodium* causing malaria finally became the mainstream explanation for the etiology of the disease, which was facilitated by both medical and socio-political factors. Differently, at the micro level, the emergence of the concepts related to “parasite” in modern China was a purer process of conceptual formulation, a result of knowledge translation.

### 4.2 The changing names of *plasmodium* and emergence of parasite

As could be observed in modern newspaper and periodical articles introducing knowledge on malaria in the early twentieth century, the early forms of several key cytological and microbiological concepts from the West, notwithstanding the immaturity of the authors’ explanation, were in the view of the Chinese readers. Though their names were not unified, those concepts participated in the translation of the Western medical concept of malaria in the following 30 years. Among them, the development and shift of concepts related to “parasite” were demonstrated by the changing terms used by the authors to denote them during that period.

Before 1910, when the theory that *Anopheles* mosquitos were the vectors of malaria was introduced to the Chinese readers, the concept of “parasite” emerged in some of the newspaper and periodical articles. Terms like “weichong,” “nueji weichong,” and “weijun” were used to denote the concept. A 1915 article entitled “nueji zhi yuanyin yu tiaozhi (etiology and treatment of malaria 瘧疾之原因與調治/疟疾之原因与调治)” demonstrated an update of the knowledge on “parasite.” In this article, Lu Yin introduced that “an’nue (按瘧/按疟; ‘an’ denoting ‘*Anopheles*’)” was an infectious disease; “jisheng dongwu (寄生動物/寄生动物; literally meaning ‘parasitical animals’)” of extremely tiny size existed in the patients’ blood, the poison of which entered the blood, causing various symptoms. As was compared with “weichong” and “weijun,” the term “jisheng dongwu” was much closer to “jishengchong (寄生蟲/寄生虫),” the contemporary Chinese name of “parasite.” The author continued to explain that the cause for malaria was a type of “weishengwu (microbe 微生物),” which was “zhi jiandan zhi dongwu (the simplest animal 至簡單之動物/至简单之动物),” and the tiny microbe could not be observed without microscope (52).

Meanwhile, the traditional view that “parasite” was a type of insect still prevailed. For instance, in “nueji zhi chuanran ji fahan zhi yuangu yu zhifa (the infection of malaria and cause and treatment for its chills 瘧疾之傳染與發寒之原故與治法/疟疾之传染与发寒之原故与治法),” Dan Lin introduced that “chong” in the blood hid in blood cells, forming a shell and fissioning in it (53). Jian Min’s “nueji zhi yuanyin yu fangyu zhiliao fa (causes for malaria and its prevention and treatment 瘧疾之原因與防禦治療法/疟疾之原因与防御治疗法)” was an example where the concepts of “bingjun (germ 病菌)” and “jishengchong” were undifferentiated. In this text, it was explained that the blood of malaria patients contained numerous germs (“wushu bingjun” 無數病菌/无数病菌), which caused the three different symptoms of the disease, the chills, fever, and sweating (54).

The failure of some newspaper and periodical articles to draw a clear line between “bacterium” and “parasite,” both as subcategories of “microbe,” with specific terms in translation was an epitome of the general situation concerning translation of scientific terminologies at the turn of the twentieth century in China, which was considered unsteady and risky (55). The unsteadiness and riskiness were further aggravated by the introduction and emergence of new terminologies. The 1918 article “nueji zhi youlai” introduced the character “sheng 笙,” which was specially coined for the concept of “parasite.” In the text, the author explained it as a type of microbe, and explained the cause for malaria as the most inferior “sheng” in the biological realm (56). Like many other characters coined to denote the newly-introduced scientific concepts, “sheng” was also left unused, owing to its elusiveness.

In “yufang nueji xinshiyan,” the earliest account of “yuansheng dongwu (原生動物/原生动物)” and “yuanchong (原蟲/原虫)” could be observed. At the beginning of the article, the author proposed the idea that the priority of malaria prevention was the elimination of mosquitos. Then, the microbes were categorized into two types, an animal-type and a plant-type; the former was called “xijun,” while the latter was called “yuansheng dongwu,” which could be abbreviated into “yuanchong”; it was believed the cause for malaria (57). An article entitled “wenchong yu nueji (mosquitos and malaria 蚊蟲與瘧疾/蚊虫与疟疾)” published in the same year in *Funyu Zazhi* (婦女雜志/妇女杂志) showed another name of *Plasmodium*, “bingchong (病蟲/病虫; literally meaning ‘worm of disease’),” in the account that the blood cells of malaria patients contained numerous tiny objects in bizarre forms, which were “bingchong” of malaria (58).

In the 1920s, though the Western etiological theory on malaria was gradually popularized among the Chinese readers, the concepts of “bacterium” and “parasite” were still not clearly differentiated. Although the terms “weijun” and “bingjun” were no longer used to denote “parasite,” some other names, such as “malaliya jun (麻拉利亞菌/麻拉利亚菌)” and “nuejun (瘧菌/疟菌),” emerged. For instance, in “nueji zhongxi zhengzhi lun (Chinese and Western treatments of malaria 瘧疾中西診治論/疟疾中西诊治论),” Huang Guocai introduced at the beginning that as had been explained by Western medical practitioners, the cause for malaria was “malaliya jun” transmitted by mosquitos into the human blood (59). In “nueji zhi zhenduan – zhiliao – bingfuzheng (diagnosis, treatment, and complication of malaria 瘧疾之診斷 – 治療 – 並副症),” Zhao Qihua denoted “malaria *Plasmodium*” by “nuejun,” and introduced its existence in human red blood cells (60).

Along with the appearance of the abovementioned terms, also in the 1920s, “nuebaozi (瘧孢子/疟孢子)” and “nuechong” emerged. The former could be seen in a 1920 article “yufang nueji fa” published in *Shen Pao*, where the author explained the etiology of malaria by stings of mosquitos (61). The latter, “nuechong,” was more commonly used in the articles published during 1920 and 1930, such as “nueji de laiyou (cause for malaria 瘧疾的來由/疟疾的来由)” (62), “nueji yu nuewen (malaria and *Anopheles* mosquitos 瘧疾與瘧蚊/疟疾与疟蚊)” (63), and “nueji tongsu qianshuo (a brief introduction to malaria for popular reading 瘧疾通俗淺説/疟疾通俗浅说)” (64).

The Chinese phrase “nueyuanchong (the term for *Plasmodium* in the contemporary Chinese language)” could be observed in introduction to the cause for malaria around 1925. For instance, Zhuang Chunzheng criticized the unorthodox explanations for the etiology of malaria, and owed the disease to “yuanchong,” of which the equivalent offered was “*Plasmodium*.” Three types of “yuanchong,” “Plasmodium Vinax (隔日瘧原蟲/隔日疟原虫),” “Plasmodium Malariae (隔二日瘧原蟲/隔二日疟原虫),” and “Plasmodium Falcipaum (夏秋瘧原蟲/夏秋疟原虫 or 惡性瘧原蟲/恶性疟原虫)” were introduced (65). It could be seen that “nueyuanchong” was first contained in specific names of different types of malaria and then used as a general term for the pathogen of the disease.

It is necessary to point out that the expressions “Plasmodium Vinax,” “Plasmodium Malariae,” and “Plasmodium Falcipaum” were used by the author in the original text. Unfortunately, “Plasmodium Vinax” was a case of misspelling; it should be “*Plasmodium vivax*.” Also, the other two types of “yuanchong” should be refined as “*Plasmodium malariae*” and “*Plasmodium falciparum*.”

Meanwhile, the terms “jishengwu (寄生物/寄生物)” and “jishengchong” successively appeared in the newspaper and periodical articles, demonstrating the gradual clarification of the concept of “parasite.” For instance, Wu Chenyi introduced that “malaria” was an infectious disease caused by “jishengwu,” which was transmitted to humans through the stings of “an’oufei wen (安歐非蚊/安欧非蚊; ‘*an’oufei*’ as another transliteration of ‘*Anopheles*’)” (66). In the same year, Yang Xingheng proposed that the pathogen of malaria was “jishengchong” (67).

The process of a number of terms for different types of *Plasmodium* emerging and the concept of “parasite,” with the use of relevant terms, being clarified in the 1920s did not help demarcate the concepts of “parasite” and “bacteria” completely. In the debate between the TCM and Western medical practitioners, the inaccurate understanding of the concepts introduced from the West by some of the TCM practitioners led to the observable linguistic fact that the terms like “weichong” and “xijun” were still used to denote “*Plasmodium*” in newspaper and periodical articles in the early 1930s. For example, in an article entitled “nueji,” the TCM practitioner Jiang Huailin stated that the Western medical practitioners owed all diseases to “xijun,” such as “malaria” featured by intermittent chills and fever, which was caused by “malaliya xijun (literally meaning bacteria of malaria 麻拉利亞細菌/麻拉利亚细菌)” (68).

After 1930, an increase of the use of terms like “yuanchong” and “jishengchong” in the dissemination of knowledge related to malaria could be observed. Yu Gongxia introduced that the entrance of “malaliya yuanchong (麻拉利亞原蟲/麻拉利亚原虫)” into the red blood cells of the human body, its gradual growth, and its reproduction through fission explained for the symptoms of malaria (69). A 1932 article entitled “malaliya bing (麻剌利亞病/麻剌利亚病; ‘malaliya’ as another form of its transliteration)” explained that the cause for malaria was “baozichong,” a primitive animal, which was also referred to as “jishengchong” (70). Shen Kangbai’s “jixing chuanran bing zhi er – nueji (acute infectious diseases, no. 2 – malaria 急性傳染病之二 – 瘧疾)” was an early account of the relation between *Plasmodium* and malaria, where the term “nueyuanchong” was used (71).

The text entitled “nueji zhi chuanran (the transmission of malaria 瘧疾之傳染/疟疾之传染)” published by *Ta Kung Pao* (大剬報/大剬报) on June 16, 1932 was an announcement on the display of an educational video about malaria by the Chinese Medical Association. The video, as was introduced by the text, explained what *Anopheles* mosquitos were, how *Plasmodium* lived in mosquito and human bodies, and how malaria was transmitted (72). The text, as an early example which brought the term “nueyuanchong” into public view, reflected the position of knowledge on malaria in the national health education at that time.

The term “nueyuanchong” was also used in “nueji” published by *Yijie Chunqiu* (醫界春秋/医界春秋), where Yuan Xiuyue introduced that the cause for malaria was claimed “yuanchong” by the Western medical practitioners, and the reason for the chills and fever suffered by the patients was the toxin generated by “nueyuanchong” entering the blood and circulating in the body (73). In the same year, a public speech delivered by Dr. Miihlens (translated differently into “米倫斯,” “茂倫思,” and “繆倫思”) on the treatment and prevention of malaria was introduced to the Chinese readers with different versions of translation. Different terms were used by different authors to denote the same concept. For instance, “*Plasmodium*” was denoted by “nueyuanchong,” “nueji yuanchong,” “nue baozi chong,” “zhangnue zhi youxing shengzhi youchong (瘴瘧之有性生殖幼蟲/瘴疟之有性生殖幼虫; ‘youxing shengzhi’ meaning ‘sexual reproduction’, and ‘youchong’ meaning ‘larvae’),” and “nuechong.”

Around 1935, as the debate between TCM and Western medicine peaked, and the relation between “nueji” and “zhangqi” was gradually clarified, the systematic knowledge on the history, etiology, symptoms, diagnosis, and prevention of malaria was further disseminated in China. Wang Shenzhi comprehensively recorded the concept of “malaria” in the Western medical history, and used terms including “jishengchong,” “nueyuanchong,” and “suzhu (宿主; the contemporary Chinese term for ‘host’)” to illustrate the mechanism of malaria transmission (74). A text in 1935 entitled “Diseases of China: Malaria,” which was written by Fames L. Maxwell, translated by Song Daren, and published by *Journal of the Medical Research Society of China* (中西醫藥/中西医药), was an early systematic account of the prevalence, distribution, etiology, pathogen, symptoms, diagnosis, prevention, and treatment of malaria in modern China. In the part concerning the etiology of the disease, the author pointed out that the pathogen of malaria was “jisheng dongwu” in the blood; it was named “nueyuanchong,” with its English equivalent “Plasmodium Malariae” presented in the parentheses, and was claimed to be transmitted to patients by “annuo feilei wenzi (安諾斐雷蚊子; ‘*annuo feilei*’ being another transliteration of ‘*Anopheles*’),” the truthfulness of which was unnecessary to be proven hereinafter, as it had been carefully explained in various types of medical writings (75).^1^

In 1936, Chen Bangxian, author of *History of Chinese Medicine*, published “nueji shi (history of malaria 瘧疾史/疟疾史),” presenting a comprehensive history of the Western and Chinese medical theories on malaria, which covered the themes including the naming of malaria in the West and China, its causes, its transmission, and its treatments. In the section about its causes, Chen introduced that in 1898, Ross discovered that “renlei nueyuanchong (the *Plasmodium* causing human malaria 人類瘧原蟲/人类疟原虫)” continued to grow in the body of mosquitos (76). In the same year, Zhu Chunlu, in “huoluan nueji liji yu xijun zhi guanxi,” introduced that “nueji xijun (瘧疾細菌/疟疾细菌; literally meaning ‘bacteria of malaria’)” was a type of “yuanchong,” and thus it was also called “nueji yuanchong”; in cases of red blood cell destruction, the toxin generated by “nueyuanchong” would flow into the human blood, circulating in the human body, and causing the chills and fever (77). The name of “xijun yuanchong” revealed that the understanding of the concept by the author was still with a traditional local tint, while the fact that the concept of “*Plasmodium*” was used as a mainstream concept to explain the etiology of malaria was clear.

## 5 “Chills and fever” and the idea of “immunity”

The translation of the concept and knowledge of “malaria” in China at the beginning of the twentieth century reflected the emergence, formulation, and establishment of some of the medical concepts from the West; meanwhile, it also reflected the absence of some of such concepts, among which “immunity,” a concept closely related to the symptoms of malaria, was a typical example. As is explained by modern medicine, the mechanism of fever is “defensive reaction” of the human body against infectious diseases, and it is caused by “pyrogens” produced by the immune system in cases of invasion of bacteria or viruses.^2^ It is evident that the malaria symptoms of “intermittent fevers” or “chills and fever,” while documented in ancient European texts dating back to the fourth and fifth centuries (16), remained inadequately explained without the theoretical framework of modern immunology. This framework appeared largely unknown in China before 1940.

Discussions on the causes for the symptoms of malaria were accessible to the Chinese readers before 1920. In the abovementioned article “nueji zhi youlai,” the author explained that the symptoms of malaria were owed to the destruction of white blood cells by “weijun” (42), mistakenly understanding the type of blood cell invaded by the pathogen of malaria. Such a wrong view on the pathogenesis of the disease was corrected in the 1910s. For instance, Lu pointed out that the pathogen causing malaria stayed in “chixuelun (赤血輪/赤血轮; an archaic name of ‘red blood cell’),” and the reason for the intermittent chills and fever suffered by the patients was still unclear; a possibility was that a type of poisonous fluid was generated when the pathogen fissioned. In other words, the symptoms of malaria were caused by the toxin of such “weishengwu” (52). Such a view on the pathogenesis of malaria that the toxin of *Plasmodium* caused the symptom of intermittent chills and fever continued to prevail in China till the 1940s.

The following examples all help prove that the concept of immunity was not established in the Chinese medical domain before 1940. Dan explained that “chong” hid in the blood cells and grew gradually into a shell, and when it left the shell, its toxin also flowed out, causing the fever of the patient (53). Shou explained that when “baozi” ruptured, its toxin flowed into the blood, and the patient would suffer seriously from the fever (61). Similarly, Yuan (73) and Zhu (77) both owed the chills and fever caused by malaria to the toxin released by *Plasmodium* circulating in the patient’s blood.

TCM, in contrast to the Western “immunity” concept, employed the concept of “qi (氣/气)” to elucidate the body’s defensive mechanisms against diseases. The proposal of “qi” as a key concept in TCM could be traced back to *Huang Di Nei Jing*, where all movements of matters were believed to be accomplished under the impetus of “qi,” a process termed “qi transformation.” Within the framework of TCM, “qi” manifests in various forms, such as “weiqi (衛氣/卫气),” “yingqi (營氣/营气),” “zongqi (宗氣/宗气),” and “xieqi (邪氣/邪气).” Among these, “weiqi,” meaning “guard qi,” or “protecting qi,” or “defensive qi,” and “yingqi,” meaning “camp qi,” both containing military terms, are considered to protect the organism and ward off intruders, thus being in charge of human body’s defensive function (21, 78). Empirical evidence suggests that qi-training (or qi-therapy), such as Qigong and Tai Chi, is beneficial in enhancing human immune function (79, 80). Given that “qi” constituted a cornerstone of the TCM theory about human body’s defense against external pathogens, the absence of Western immunological framework in explaining malaria’s pathology during the studied period appeared less conspicuous.

## 6 Discussion and conclusion

Through systematic analysis of lexical entries for “malaria” and “ague” in English-Chinese dictionaries (1822–1949) and discourses about malaria in late Qing and Republican-era newspapers and periodicals, this study identifies the 1940s as the critical juncture when “nueji” solidified as the standardized Chinese equivalent for malaria. This lexical stabilization coincided with the institutionalization of western etiological frameworks—notably the assimilation of nueyuanchong (*Plasmodium*) and jishengchong (parasite) into Chinese medical discourse. Nevertheless, the absence of “immunity” as an explanatory concept underscores the selective nature of cross-cultural knowledge transfer, revealing epistemic boundaries in China’s medical modernization.

The findings demonstrate that translating ancient disease concepts transcends mere terminological substitution; it constitutes a tripartite negotiation wherein textual practices (e.g., the symptom-focused translations of “ague” in missionary dictionaries) initially localized foreign concepts, discursive contests in newspapers and periodicals mediated public acceptance of Western etiology versus traditional beliefs, and institutional forces (e.g., the terminology guidelines) finally codified biomedical paradigms. This dynamic negotiation—spanning lexicons, public discourses, and institutional power—ultimately catalyzed the epistemological foundations of Chinese medical modernity.

The narrative approach adopted by this article tends to obscure the individual characteristics of the recipients and promoters of Western medical knowledge in China in the examined period. In other words, the challenges of information exchange, or the complexities of knowledge translation, has not been thoroughly explored. On the side of the recipients, their level of education or literacy was not taken into account. Consequently, the efficacy of science popularization during the studied period remains unclear. It could be possible that such efficacy was hindered by the ability to access and understand written materials about Western medicine, as existing research has demonstrated that the literacy rate of Chinese people in the studied period was not high. According to Rawski’s seminal work, the functional literacy rate among Chinese males in the Qing period was estimated to be between 30 and 45%, while that for Chinese females was only between 2 and 10% (81). The literacy rate of Chinese people in the Republican period was not significantly improved, as Buck’s comprehensive rural surveys in the early 1930s reported a male literacy rate of 30.3% and a female literacy rate of 1.2% (82). The significant gender and urban–rural disparities make it even more complicated to discuss the reception and perception of Western scientific knowledge by the Chinese readers. As a result, a large room for further investigation concerning the recipients of Western medical concepts in China emerges.

On the side of the promoters, individual differences were also pronounced. Those who promoted Western medical knowledge exhibited significant variation in education background, profession, purpose, and strategy, endowing the medical discourse of the studied period with a distinctly “heteroglossic” character (83). Different promoters, with their unique backgrounds and objectives, demonstrated diverse approaches to translating, introducing and promoting Western medical concepts. Some underscored the scientific aspects of Western medicine, emphasizing the differences between the introduced knowledge and the traditional beliefs. In such cases, transliteration may be selected as a method of term translation. Others highlighted its practical utility, thereby advancing the localization of relevant knowledge. Conversely, misinterpretations might also be observed, as is illustrated by the cases of “Plasmodium Vinax” and “banwen” observed and mentioned above in this article. These varied approaches would have inevitably influenced the recipients’ reception and perception of the introduced knowledge. However, the present study does not sufficiently analyze such differences; instead, the translation, introduction, and promotion of Western medical knowledge on malaria have been viewed as “a result of joint effort” made by the different promoters (83). Consequently, the complexities concerning the promoters are inadequately explored. This limitation suggests a need for further research to scrutinize how these individual differences among promoters impacted the dissemination of Western medical knowledge in China, which would provide a more nuanced understanding of the dynamics underlying the promotion and reception of Western medicine in the modern Chinese context.

Presently and globally, malaria remains a significant public health concern. According to the World Health Organization (WHO), in 2023, there were approximately 263 million new malaria cases across 83 countries and 597,000 deaths worldwide; the African Region bore 95% of these deaths (84). While artemisinin-based combination therapy (ACT) is effective, the aim of The Global Technical Strategy for Malaria 2016–2030 (GTS) to reduce malaria case incidence by 90% from the 2015 baseline by 2030 is unlikely to be achieved (85).

China, as one of the earliest countries to understand malaria, achieved a remarkable milestone when it was certified malaria-free by WHO in 2021 (86). Historically, malaria was prevalent in China. In 2010, in response to the global malaria eradication initiative, the Chinese government launched the “National Action Plan for Malaria Elimination,” prioritizing enhanced blood tests, early diagnosis, free mass distribution of long-lasting insecticide-treated nets (LLINs), health education, and real-time web-based surveillance; innovatively, in the elimination stage, China adopted an adaptive case- and focus-oriented strategy, the “1-3-7” approach (1-day reporting, 3-day investigation, 7-day response), and constructed elimination-specific reporting systems (87). Malaria elimination in China represents not an endpoint, but a sustained effort to mitigate imported malaria incidences, as well as to advance global health collaboration by disseminating China’s understanding of malaria (86). Part of such understanding was deeply rooted in the nation’s 3,000-year epidemiological history accounted in this study.

## Footnotes

^1^The first version of this article was translated by Song Ze and published in *Periodicus Medico-pharmaceuticus*. In this version, “Plasmodium Malariae” was mistakenly translated into “sanri nueyuanchong 三日瘧原蟲 (a type of plasmodium).” The 1935 version corrected this mistake, showing an update of the malaria-related knowledge.

^2^This definition was retrieved from: https://www.britannica.com/science/fever.

## References

[1] Li WJ. The dynamics of translation studies: interdisciplinary perspectives and approaches. Transl Interpreting Stud. (2017) 12:355–66. doi: 10.1075/tis.12.2.08wen

[2] Vandepitt S Lefever E. Translation as a multilingual activity in the digital era. Rev Fr Linguist Appl. (2018) 23:59–72. Available at: https://shs.cairn.info/journal-revue-francaise-de-linguistique-appliquee-2018-2-page-59?lang=en

[3] Rędzioch-Korkuz A. Towards a semiotic model of interlingual translation. Semiotica. (2020) 236:215–30. doi: 10.1515/sem-2019-0027

[4] Rundle C. Theories and methodologies of translation history: the value of an interdisciplinary approach. Translator. (2014) 20:2–8. doi: 10.1080/13556509.2014.899090

[5] O’Sullivan C. Introduction: rethinking methods in translation history. Transl Stud. (2012) 5:131–8. doi: 10.1080/14781700.2012.663594

[6] Olohan M. History of science and history of translation: disciplinary commensurability? Translator. (2014) 20:9–25. doi: 10.1080/13556509.2014.899091

[7] Zou ZH. The history of Chinese translation in the twentieth century and the expansion of new field of modern history. Hebei Acad J. (2019) 39:63–72.

[8] Asen D. “Manchu anatomy”: anatomical knowledge and the Jesuits in seventeenth-and eighteenth-century China. Soc Hist Med. (2009) 22:23–44. doi: 10.1093/shm/hkn097

[9] Bosmia A Bosmia AN Patel TR Watanabe K Shoja MM Loukas M. Benjamin Hobson (1816–1873): his work as a medical missionary and influence on the practice of medicine and knowledge of anatomy in China and Japan. Clin Anat. (2014) 27:154–61. doi: 10.1002/ca.22230, PMID: 23553744

[10] Andrews B. Ding Fubao and the morals of medical modernization. East Asian Sci Technol Med. (2015) 42:7–38. doi: 10.1163/26669323-04201002

[11] Andrews B. Tuberculosis and the assimilation of germ theory in China, 1895—1937. J Hist Med Allied Sci. (1997) 52:114–57. 9071849 10.1093/jhmas/52.1.114

[12] Chang CF. The localization of the cowpox vaccination in early nineteenth-century China. Bull Inst Hist Philol Acad Sin. (2007) 78:755–812.

[13] Tian XL. (2011). Relocating science: Medical missions and Western medicine in nineteenth-century China [dissertation]. Chicago: University of Chicago.

[14] Yu XZ Chen SY. Between medicine and social-culture: 100 years studies of medical history in the qing dynasty. J Cent China Normal Univ (Humanit Soc Sci). (2017) 3:111–28.

[15] Saleem S Bianucci R Galassi FM Nerlich AG. Editorial: ancient diseases and medical care: paleopathological insights. Front Med. (2023) 10:1140974. doi: 10.3389/fmed.2023.1140974 PMC9936186 36817765

[16] Boualam MA Pradines B Drancourt M Barbieri R. Malaria in Europe: a historical perspective. Front Med. (2021) 8:691095. doi: 10.3389/fmed.2021.691095, PMID: 34277665 PMC8277918

[17] Neghina R Neghina AM Marincu I Iacobiciu I. Malaria, a journey in time: in search of the lost myths and forgotten stories. Am J Med Sci. (2010) 340:492–8. doi: 10.1097/MAJ.0b013e3181e7fe6c, PMID: 20601857

[18] Cunha CB Cunha BA. Brief history of the clinical diagnosis of malaria: from Hippocrates to Osler. J Vector Borne Dis. (2008) 45:194–9. 18807375

[19] Burke PF. Malaria and Greco-Roman world: a historical and epidemiological survey. In: Haase W, editor. Band 37/3. Teilband Philosophie, Wissenschaften, Technik. Wissenschaften (Medizin und Biologie [Forts.]). Berlin/New York: De Gruyter (1996). 2252–81.

[20] Anderson D. Dorland’s illustrated medical dictionary. 32nd ed. Philadelphia: Elsevier (2012).

[21] Unschuld PU Tessenow H (Trans). Huang Di Nei Jing Su wen: An annotated translation of Huang Di’s inner classic – Basic questions (1). Berkeley/Los Angeles: University of California Press (2011).

[22] Fan XZ. A new history of diseases in China. Beijing: Chinese Medical Classics Press (1989).

[23] Hsu E. The history of qing Hao in the Chinese materia medica. Trans R Soc Trop Med Hyg. (2006) 100:505–8. doi: 10.1016/j.trstmh.2005.09.020, PMID: 16566952

[24] Hsu E. Reflections on the “discovery” of the antimalarial qinghao. Br J Clin Pharmacol. (2006) 61:666–70. doi: 10.1111/j.1365-2125.2006.02673.x, PMID: 16722826 PMC1885105

[25] Morrison R. A dictionary of the Chinese language in three parts. Macau: The Honorable East India Company's Press (1822).

[26] Medhurst W. English and Chinese dictionary. Shanghai: The Mission Press (1847).

[27] Lobscheid W. English and Chinese dictionary with the Punti and mandarin pronunciation. Hong Kong: Daily Press Office (1866–1869).

[28] Doolittle J. Vocabulary and hand-book of the Chinese language. Fuzhou: Rozario, Marcal and Company (1872).

[29] Mateer W. Technical terms. Shanghai: American Presbyterian Mission Press (1904).

[30] Yen WC. An English and Chinese standard dictionary. Shanghai: Commercial Press (1908).

[31] Hemeling K. English-Chinese dictionary of the standard Chinese spoken language and handbook for translators. Shanghai: Statistical Department of the Inspectorate General of Customs (1916).

[32] Wilhelm R. Deutsch-Englisch-Chinesisches Fachwörterbuch. Qingdao: Deutsch-Chinesischen Hochschule (1911).

[33] Bianyi C Hui W. English-Chinese modern medical dictionary. Hangzhou: Xinyi Shuju (1949).

[34] Anonymous. Etiology of malaria and scabies. Church News. (1869) 29:10.

[35] Anonymous. Zhangqibing is malignant nueji. The central daily news (1935) 9, 16–17.

[36] Anonymous. Guangxi government confirmed that zhangqi is malignant nueji. Peipinger Medizinische Monatsschrift. (1936) 4:41.

[37] Anonymous. Malaria. Social Welfare Tientsin Reports. (1927) 14:06–20.

[38] Du H. Nueji-like diplomacy. Xin Wen Bao. (1929) 4:10–21.

[39] Arrow KJ Panosian CB Gelband H. Saving lives, buying time: Economics of malaria drugs in an age of resistance. Washington, D.C.: The National Academies Press (2004). 25009879

[40] Nosten F Richard-Lenoble D Danis M. A brief history of malaria. Presse Med. (2022) 51:104130. doi: 10.1016/j.lpm.2022.104130, PMID: 35667599

[41] Anonymous. Relation between malaria and mosquitos. Chinese Christian Intelligencer. (1908) 291:7.

[42] Wang YF. Origin of malaria. Nong Gong Shang Bao. (1908) 43:45.

[43] Silva NM Santos NC Martins IC. Dengue and Zika viruses: epidemiological history, potential therapies, and promising vaccines. Trop Med Infect Dis. (2020) 5:150. doi: 10.3390/tropicalmed5040150, PMID: 32977703 PMC7709709

[44] Shen ZG. Is malaliya really the pathogen of nueji? Yixue Zazhi. (1929) 35:11–2.

[45] Ji H. Malaria. Zhongguo Yixue Yuekan. (1928) 1:30–2.

[46] Zhu ZS. Self-treatment of malaria. Changshou Zhoukan. (1933) 1:7.

[47] Anonymous. A brief introduction to malaria. Yixue Zhoukan Ji. (1932) 6:125–8.

[48] Yao J. Refuting the fallacy that “malaria is transmitted through air and food”. Minzhong Yiyao Huikan. (1934) 1:76.

[49] Huang ZS. The similarities in malaria pathology discussed by TCM and western medicine. Ziqiang Yikan. (1929) 8:5

[50] Wu G. Chinese and western views on malaria. Shanghai Med Week. (1930) 42:411–2.

[51] Zhang ZH. A brief account of malaria. Yi Xue Zazhi. (1930) 57:64–6.

[52] Lu Y. Etiology and treatment of malaria. Qingnian. (1915) 18:197–9.

[53] Dan L. The infection of malaria and cause and treatment for its chills. Chin Christ Intell. (1917):712–69.

[54] Jian M. Causes for malaria and its prevention and treatment. Qingnian Jinbu. (1918) 18:66–8.

[55] Amelung I. Zhenshi yu Jiangou: Zhongguo Jindaishi ji Kejishi Xintan (truth and construction: New explorations in modern Chinese history and the history of science). Beijing: Social Sciences Academic Press (2019).

[56] Chen HL. The origin of malaria. Huazhong Cui. (1918) 1:83.

[57] Yun H. A new test on malaria prevention. Sin Min Pao. (1919) 6:17–8.

[58] Anonymous. Mosquitos and malaria. Funyu Zazhi. (1919) 5:1–10.

[59] Huang GC. Chinese and western treatments of malaria. Shaoxing Yiyao Xuebao. (1920) 10:46–7.

[60] Zhao QH. Diagnosis, treatment, and complication of malaria. Tongde Yixue. (1922) 3:1–8.

[61] Shou G. Methods of malaria prevention. Shen Pao. (1920) 7:18.

[62] Wu DJ. Cause for malaria. Shaonian. (1922) 12:5–10.

[63] Rao Z. Malaria and anopheles mosquitos. Shen Pao. (1922) 20:10–8.

[64] Anonymous. A brief introduction to malaria for popular reading. Tiande Yiliao Xinbao. (1927) 1:1–6.

[65] Zhuang CZ. On malaria and its prevention. Kwang Chi Med J. (1924) 1:55–7.

[66] Wu CY. Malaria. Kwang Chi Med J. (1928) 5:57–8.

[67] Yang XH. Identifying the causes for malaria, diphtheria, and syphilis before treatment. Yi Xue Zazhi. (1928) 46:45–6.

[68] Jiang HL. Malaria. Shijie Yibao. (1930) 1:151.

[69] Yu GX. On malaria. Ziqiang Yikan. (1921) 15:38–44.

[70] Ding HK. Malaliya bing. Modern Home. (1932) 1:64–6.

[71] Shen KB. Acute infectious diseases, no. 2 – malaria. Periodicus Medico-Pharmaceuticus. (1932) 86:29–36.

[72] Anonymous. The transmission of malaria. Ta Kung Pao. (1932) 6:5.

[73] Yuan XY. Malaria. Yijie Chunqiu. (1933) 78:13.

[74] Wang SZ. History of malaria. Neochrome. (1934) 21:30–6.

[75] Maxwell F. Diseases of China: malaria. J Med Res Soc China. (1935) 1:96–114.

[76] Chen BX. History of malaria. Yishi Gonglun. (1936) 4:4–15.

[77] Zhu CL. The relationship between cholera, malaria, dysentery, and bacteria. Wujiang Guoyi Xuebao. (1936) 3:4–5.

[78] Xia FF (2019). A comparative study of defensive qi in traditional Chinese medicine and immunity in western medicine [master’s thesis]. Qingdao: Qingdao university

[79] Lee MS. Effects of qigong on immune cells. Am J Chin Med. (2009) 37:421–9. doi: 10.1142/S0192415X03001016 12856872

[80] Jahnke R Larkey L Rogers C Etnier J Lin F. A comprehensive review of health benefits of qigong and tai chi. Am J Health Promot. (2010) 24:e1–e25. doi: 10.4278/ajhp.081013-LIT-248, PMID: 20594090 PMC3085832

[81] Rawski ES. Education and popular literacy in Ch’ing China. Ann Arbor: University of Michigan Press (1979).

[82] Buck JL. Land utilization in China. New York: Paragon Book Reprint Corp (1964).

[83] Miao P. Diabetes: a transcultural history of a disease concept in the late Qing and republican China. Chin Med Cult. (2023) 6:247–57. doi: 10.1097/MC9.0000000000000072

[84] World Health Organization. World malaria report 2024: Addressing inequality in the global malaria response. Geneva: World Health Organization (2024).

[85] World Health Organization. World health statistics 2025: Monitoring health for the SDGs, sustainable development goals. Geneva: World Health Organization (2025).

[86] Zhou XN. China declared malaria-free: a milestone in the world malaria eradication and Chinese public health. Infect Dis Poverty. (2021) 10:98. doi: 10.1186/s40249-021-00882-9, PMID: 34253259 PMC8276478

[87] Huang F Feng XY Zhou SS Tang LH Xia ZG. Establishing and applying an adaptive strategy and approach to eliminating malaria: practice and lessons learnt from China from 2011 to 2020. Emerg Microbes Infections. (2022) 11:314–25. doi: 10.1080/22221751.2022.2026740, PMID: 34989665 PMC8786258

